# Differential induction of muscle atrophy pathways in two mouse models of spinal muscular atrophy

**DOI:** 10.1038/srep28846

**Published:** 2016-06-28

**Authors:** Marc-Olivier Deguise, Justin G. Boyer, Emily R. McFall, Armin Yazdani, Yves De Repentigny, Rashmi Kothary

**Affiliations:** 1 Regenerative Medicine Program, Ottawa Hospital Research Institute, Ottawa, Ontario, K1H 8L6 Canada; 2 Department of Cellular and Molecular Medicine, University of Ottawa, Ottawa, Ontario, K1H 8M5 Canada; 3 Centre for Neuromuscular Disease, University of Ottawa, Ottawa, Ontario, K1H 8M5 Canada; 4 Department of Medicine, University of Ottawa, Ottawa, Ontario, K1H 8M5 Canada

## Abstract

Motor neuron loss and neurogenic atrophy are hallmarks of spinal muscular atrophy (SMA), a leading genetic cause of infant deaths. Previous studies have focused on deciphering disease pathogenesis in motor neurons. However, a systematic evaluation of atrophy pathways in muscles is lacking. Here, we show that these pathways are differentially activated depending on severity of disease in two different SMA model mice. Although proteasomal degradation is induced in skeletal muscle of both models, autophagosomal degradation is present only in *Smn*^*2B*/−^ mice but not in the more severe *Smn*^*−/−*^*; SMN2* mice. Expression of FoxO transcription factors, which regulate both proteasomal and autophagosomal degradation, is elevated in *Smn*^*2B*/−^ muscle. Remarkably, administration of trichostatin A reversed all molecular changes associated with atrophy. Cardiac muscle also exhibits differential induction of atrophy between *Smn*^*2B*/−^ and *Smn*^*−*/*−*^*; SMN2* mice, albeit in the opposite direction to that of skeletal muscle. Altogether, our work highlights the importance of cautious analysis of different mouse models of SMA as distinct patterns of atrophy induction are at play depending on disease severity. We also revealed that one of the beneficial impacts of trichostatin A on SMA model mice is via attenuation of muscle atrophy through reduction of FoxO expression to normal levels.

Spinal muscular atrophy (SMA) is a childhood neuromuscular genetic disease affecting 1 in 6,000 to 10,000 live births^1^,^2^. SMA pathological hallmarks include motor neuron loss and severe muscle atrophy of the limb and trunk muscles. In 1995, the disease-causing gene, survival motor neuron 1 (*SMN1*), was identified^3^. A mutation or deletion in the *SMN1* gene impairs SMN protein production. A second nearly identical copy of the gene, *SMN2*, is present at the same locus as *SMN1*^3^. However, a single base pair substitution in *SMN2* profoundly limits its ability to produce full length SMN protein^4^. Thus, increasing *SMN2* copy number leads to phenotypes of reduced severity that can be classified on a spectrum (reviewed in^5^).

The mouse only harbors a single *Smn* gene and homozygous loss is embryonically lethal^6^. The addition of a human *SMN2* transgene to *Smn*^*−*/*−*^ mice yielded the original mouse model of SMA (*Smn*^*−*/*−*^*; SMN2;* also referred to as the severe model), which presents with a severe phenotype and lives to a maximum of postnatal day (P) 6^7^. Recently, mouse models of SMA with less severe phenotypes have been generated in an effort to uncover novel disease mechanisms and to test several therapeutic approaches. One of these is the *Smn*^*2B*/−^ mouse model. Instead of incorporating the human *SMN2* transgene, this mouse harbours a 3 nucleotide substitution in the exonic splice enhancer of exon 7 (2B mutation) in one allele of the mouse *Smn* gene, while the other allele is null^8^,^9^. Consequently, the mice present with a phenotype associated with a longer life span (~P30) and enhanced motor function relative to the *Smn*^*−*/*−*^*; SMN2* mouse model^9^.

It has always been assumed that muscle defects observed in SMA were completely attributable to degenerating motor neurons. However, recent work on both the *Smn*^*−*/*−*^*; SMN2* and *Smn*^*2B*/−^ model mice revealed robust intrinsic muscle weakness prior to any overt motor neuron pathology^10^. Muscle weakness in these models was attributed to impaired muscle development supported by the aberrant expression of several proteins involved in myogenesis^10^,^11^,^12^. Atrophy, a direct consequence of motor neuron loss, is likely a contributing factor to muscle weakness, but it has not been systematically studied in SMA. Different atrophy initiating stimuli, such as fasting, denervation, and other systemic catabolic states activate common transcriptional changes in the atrogenes^13^,^14^. Myogenin (MyoG) is a transcription factor known to induce E3 ligases important in proteolytic sarcomere breakdown. However, the MyoG pathway becomes activated only in neurogenic atrophy^15^,^16^. This pathway has been previously investigated in the *Smn*Δ*7* model mice (*Smn*^*−*/*−*^*; SMN2*^+/+^*; Smn*Δ*7*^+/+^) and SMA patients where increased myogenin expression correlated with increased expression of Muscle Ring Finger 1 (MuRF1, TRIM63) and Atrogin-1 (MAFbx, Fbxo32) in skeletal muscles^17^. Importantly, myogenin-dependent increases in atrogene levels could be attenuated by treating *Smn*Δ*7* mice with the pan-histone deacetylase inhibitor trichostatin A (TSA), possibly due to its proposed effect on histone deacetylase 4 (HDAC4)^17^. TSA has also been proposed to increase SMN expression through *SMN2* transcriptional modulation^18^, which in turn could halt atrophy. However, other studies have suggested that the beneficial effects of TSA are not through SMN induction^19^. Thus, it remains unclear whether TSA functions in a SMN-dependent or independent manner to reverse atrophy in the *Smn*Δ*7* model. Other studies have examined the contribution of MuRF1 and Atrogin-1 in SMA by crossing *Smn*Δ*7* mice with MuRF1 or Atrogin-1 null animals^20^. The survival time and body weight of these mice matched the non-transgenic *Smn*Δ*7* mice control mice, suggesting MuRF1 or Atrogin-1 are not the only players in atrophy and that other pathways may be involved^20^. Assessment of autophagy or other potential triggers to the degradation machinery have not been investigated in the context of SMA and might provide answers to this conflicting evidence.

Unlike MyoG, FoxO transcription factors are implicated in a much wider variety of atrophy types, including neurogenic atrophy, where it is thought to initiate but also maintain atrophy^14^,^21^. Additionally, FoxO proteins induce the expression of proteasomal genes, such as MuRF1 and Atrogin-1, as well as autophagic genes^21^,^22^,^23^. This dual pathway control is important since autophagy has been reported to contribute to atrophy upon denervation^21^,^22^,^23^. Hence, FoxO transcription factors are thought to control half of the genes identified in the molecular “common atrophy blueprint” present in different atrophy types^21^,^22^.

Here, we have investigated the potential contribution of autophagy and its regulation in the context of SMA. In addition, we have assessed whether or not SMA severity affects atrophy molecular profiles in two SMA model mice for which atrophy characterization is lacking. We were further interested in deciphering the mechanisms by which TSA protects against atrophy using the *Smn*^*2B*/−^ model mice, which does not harbor the human *SMN2* transgene. Lastly, we extended our analysis to cardiac muscle, which has been previously described to have smaller caliber fibers^24^.

## Results

### Atrophy in skeletal muscles from *Smn* ^*−*/*−*^ *; SMN2* mice is marked by increased proteasomal degradation without signs of autophagosomal protein breakdown

**Figure 1:**
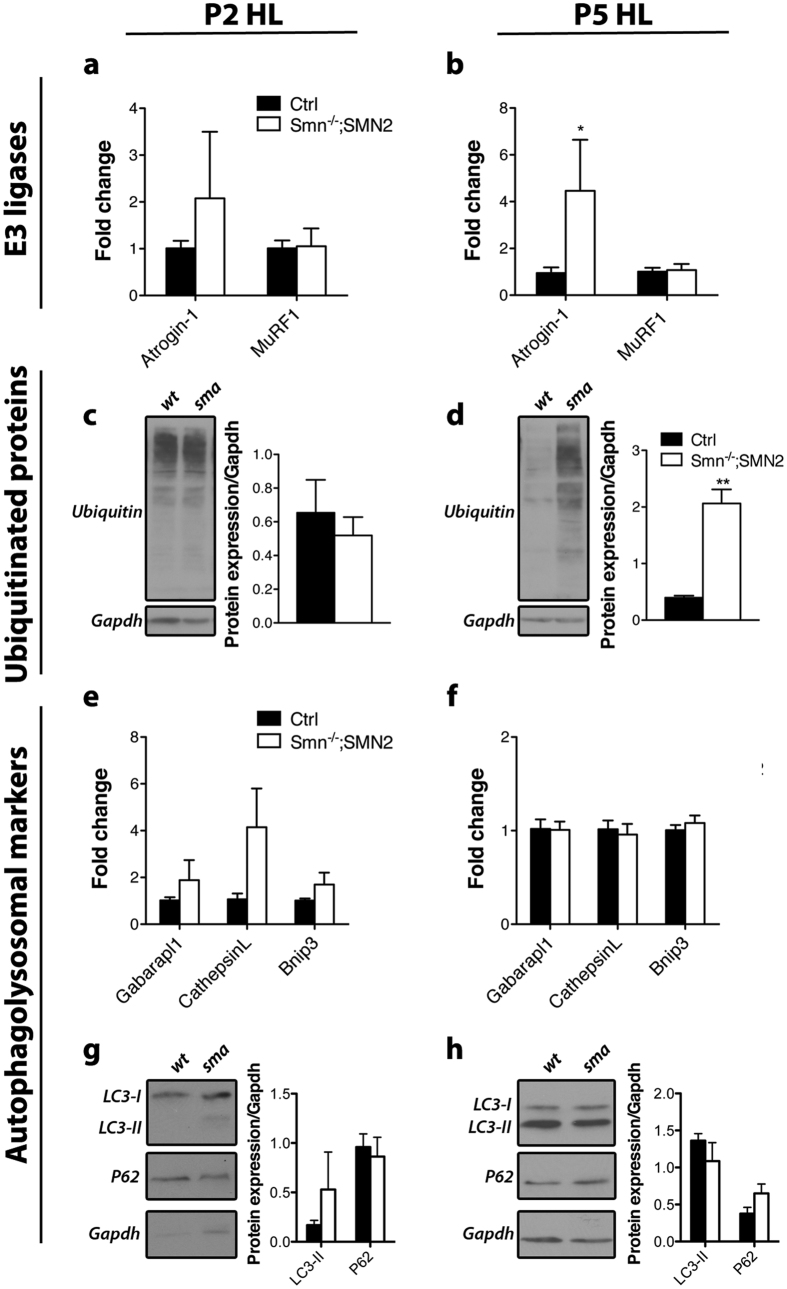
Characterization of the modes of skeletal muscle atrophy in *Smn*^*−*/*−*^*; SMN2* mice. (**a,b**) Levels of Atrogin-1 and MuRF1 mRNA are not significantly changed at pre-symptomatic stage (P2) but Atrogin-1 is increased at symptomatic stage (P5) in the hindlimb (HL) of *Smn*^*−*/*−*^*; SMN2* mice (p = 0.021). (**c,d**) Levels of ubiquitinated proteins are unchanged at P2 but are significantly increased at P5 (p = 0.0061). (**e,f**) Gabarapl1, CathepsinL and Bnip3 transcript levels are unchanged at both P2 and P5 in muscles of *Smn*^*−*/*−*^*; SMN2* mice. (**g,h**) No change was detected in LC3-II and P62 protein levels at both time points in hindlimb muscles of *Smn*^*−*/*−*^*; SMN2* mice. (N = 4 for a-g, N = 3 for h; *p ≤ 0.05).

MuRF1 and Atrogin-1 are thought to be the main E3 ligases involved in proteasomal protein breakdown of muscles^25^,^26^. We decided to focus on the expression of these E3 ligase genes since atrophy is a transcription dependent process^21^. Moreover, protein levels of E3 ligases and of autophagosome-lysosome related proteins are not always representative in wasting muscles^27^. Assessment of the mRNA levels for these genes in pre-symptomatic P2 *Smn*^*−*/*−*^*; SMN2* hindlimb muscles revealed no difference from control muscles (Fig. 1a). In contrast, increase in transcripts for Atrogin-1 was observed in *Smn*^*−*/*−*^*; SMN2* hindlimb muscles at P5 (p = 0.021) (Fig. 1b), concordant with motor neuron pathology at this age^7^. However, MuRF1 transcript expression was unchanged (Fig. 1b). Interestingly, the temporal expression increase of MuRF1 mRNA is much slower and less drastic than Atrogin-1 in fully denervated muscles^14^, which might explain the lack of a measured change in the partially denervated muscles from the *Smn*^*−*/*−*^*; SMN2* mice. The main function of E3 ligases is ubiquitination of proteins to direct them for proteasomal degradation. We therefore investigated the ubiquitination status of proteins to confirm the biological relevance of the increased expression of Atrogin-1 transcripts. Accordingly, we observed a significant elevation in the level of ubiquitinated proteins in *Smn*^*−*/*−*^*; SMN2* hindlimb muscles at P5 (p = 0.0061) (Fig. 1d), but not at P2 (Fig. 1c).

Autophagosomal and lysosomal processes have been reported in biopsies from patients that had been clinically diagnosed as having SMA^28^,^29^. However, autophagosomal implications in SMA preclinical models have not been established. We first investigated the expression of important contributors of these pathways and observed relatively unchanged mRNA levels for GABA(A) Receptor-Associated Protein Like 1 (Gabarapl1), CathepsinL and BCL2/adenovirus E1B 19 kDa protein-interacting protein 3 (Bnip3) in P2 and P5 hindlimb muscles when compared to controls (Fig. 1e,f). We next examined protein levels of sequestosome-1 (sqstm1 or P62) and post-translational forms of microtubule-associated protein light chain 3 beta (MAP1LC3B or LC3). LC3 is the most commonly used measure to identify autophagosome formation^30^. It is required for the elongation of the phagophore and LC3 lipidation from LC3-I to LC3-II is required for its function^31^. P62 is important in mediating breakdown of protein aggregates by binding to LC3-II^31^,^32^. We observed that the double lipidated form of LC3 was not elevated in P2 or P5 *Smn*^*−*/*−*^*; SMN2* hindlimb muscles (Fig. 1g,h). Similarly, P62 was unchanged (Fig. 1g,h). Altogether, these results confirm that proteasomal degradation is induced while autophagic processes are not contributing to atrophy in muscles of the severe *Smn*^*−*/*−*^*; SMN2* model mice.

### Muscle atrophy in *Smn* ^*2B*/−^ mice involves both proteasomal and autophagosomal protein breakdown

**Figure 2:**
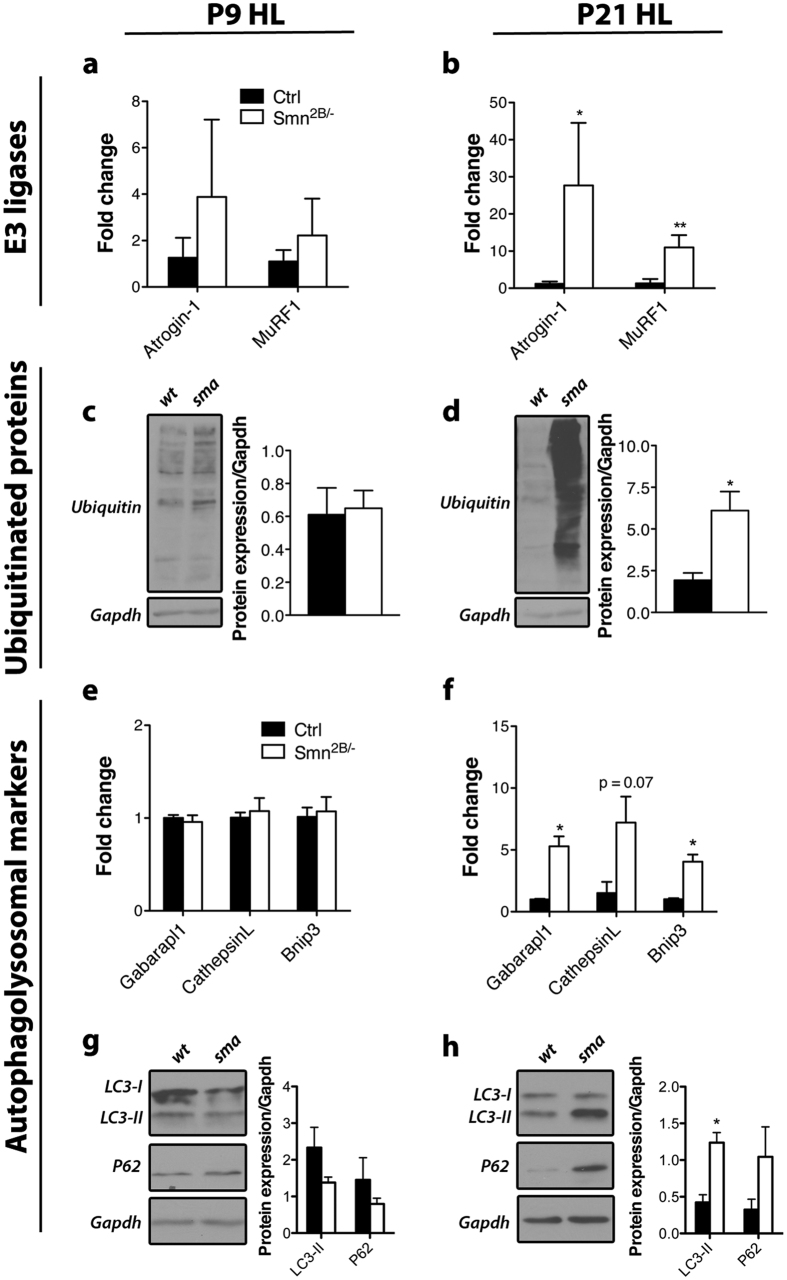
Characterization of the modes of skeletal muscle atrophy in *Smn*^*2B*/−^ mice. (**a,b**) Levels of Atrogin-1 and MuRF1 mRNA are not significantly changed at pre-symptomatic stage (P9) but are increased at symptomatic stage (P21) in the hindlimb (HL) of *Smn*^*2B*/−^ mice (p = 0.05 and 0.0067 respectively). (**c,d**) Levels of ubiquitinated proteins are unchanged at P9 but are significantly increased at P21 (p = 0.028). (**e,f**) Gabarapl1, CathepsinL and Bnip3 transcript levels are unchanged at pre-symptomatic stage (P9) but are significantly elevated at P21 in hindlimb muscles (p = 0.012, 0.066, and 0.012, respectively). (**g,h**) Protein expression of LC3-II and P62 is relatively unchanged at pre-symptomatic stage and LC3-II is significantly increased at symptomatic stage in hindlimb muscles of *Smn*^*2B*/−^ mice (p = 0.011). (N = 4 for a-f, N = 3 for g-h; p ≤ 0.05 for * and p ≤ 0.01 for **).

The heterogeneity of severity in SMA could potentially have differing molecular underpinnings given the variability in onset of the disease and its progression. Noteworthy, other groups have reported that this is likely to occur but evidence has so far been lacking^33^. Therefore, we analysed the less severe *Smn*^*2B*/−^ model to compare to the data from the *Smn*^*−*/*−*^*; SMN2* mouse (see above). Interestingly, the *Smn*^*2B*/−^ mice showed a different molecular atrophy signature compared to the severe model. Atrogin-1 and MuRF1 mRNA levels showed a significant increase (p = 0.05 and 0.0067, respectively) in P21 post-symptomatic but not in P9 pre-symptomatic hindlimb muscles (Fig. 2a,b). This increase of E3 ligases transcripts at P21 was accompanied by increased protein ubiquitination in hindlimb muscles of P21 *Smn*^*2B*/−^ mice (p = 0.028) (Fig. 2c,d).

Next, we examined expression of the autophagosomal markers. Strikingly, Gabarapl1, CathepsinL and Bnip3 transcripts were all induced in hindlimb muscle at symptomatic stage (p = 0.012, 0.066, and 0.012 respectively) but not at pre-symptomatic stage (Fig. 2e,f). These changes were accompanied by a significant increase in LC3-II protein levels only at P21 (p = 0.011) (Fig. 2g,h). A trend toward increased P62 levels at P21 was also observed (Fig. 2h). Even though levels of P62 usually increase when autophagic flux is blocked, it has also been observed during increased autophagic flux^30^. The latter is more likely occurring in SMA atrophying muscle. Altogether, these results point towards participation of autophagic protein degradation in muscles of *Smn*^*2B*/−^ mice but not *Smn*^*−*/*−*^*; SMN2* mice.

**Figure 3:**
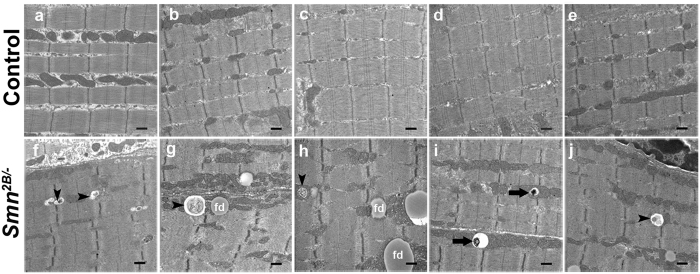
Ultrastructural analysis of TA muscles from *Smn*^*2B*/−^ mice reveals an increase in autophagic vacuoles. (**a–e**) Representative micrographs (20,000x) of *Smn*^*2B*/+^ TA muscles show that autophagic vacuoles (autophagosomes and autolysosomes) are not detectable. (**f–j**) Representative micrographs (20,000x) of *Smn*^*2B*/−^ TA muscles show several examples of autophagic vacuoles with various degrading cellular structures (black arrowheads) or electron dense material (black arrows). These vacuoles of different size can be observed adjacent to Z-discs and mitochondria in skeletal muscle. A large number of fat droplets (fd) were also identified in the *Smn*^*2B*/−^ TA muscles. (N = 3 for all experiments). Scale bar = 500 nm.

To further confirm that autophagy is induced in the *Smn*^*2B*/−^ model, we performed ultrastructural analysis of the tibialis anterior (TA) muscle. We analysed muscles from 3 individual mice, two of which showed more extensive vacuolization. We expected some variability since not all fibers are denervated in SMA muscles. Vacuoles were often observed containing degraded cytoplasmic content, mitochondria or electron dense material, highly suggestive of autophagy-like processes (Fig. 3). Moreover, structures observed closely resemble those seen in muscles co-transfected with constitutively active FoxO3^23^. Similar structures were also previously noted in ultrastructural analysis of clinically diagnosed SMA patient muscles^28^,^29^. We have also observed a large number of vacuoles that appear similar to fat droplets (e.g. see Fig. 3h)^34^. In association with the molecular data, we conclude that autophagy is playing a role in the atrophying muscles of *Smn*^*2B*/−^ mice.

### TSA administration to *Smn* ^*2B*/−^ mice reverses the expression of atrophic markers

**Figure 4:**
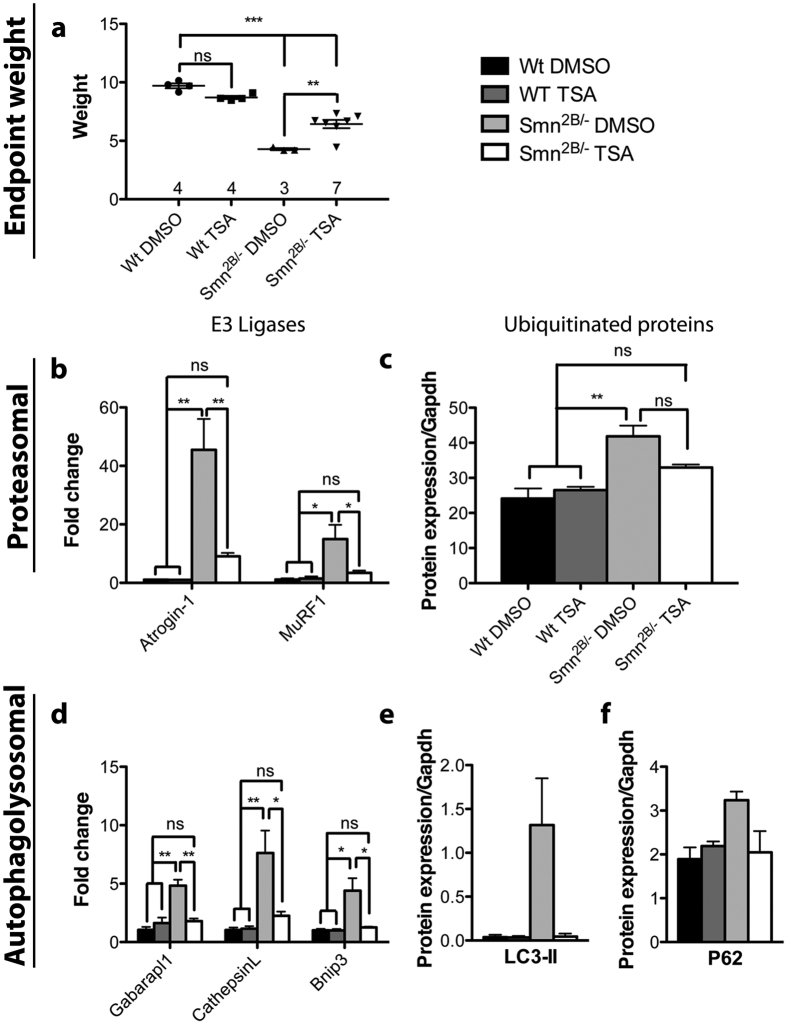
TSA administration in *Smn*^*2B*/−^ mice reversed both proteasomal and autophagosomal atrophy. (**a**) Mice were treated with either DMSO or TSA daily from P3 to P21, and then sacrificed for analysis. DMSO-treated *Smn*^*2B*/−^ mice were significantly smaller in weight compared to control mice (p ≤ 0.001). TSA-treated mutant mice showed a significant increase in weight compared to DMSO-treated *Smn*^*2B*/−^ mice (p ≤ 0.01), although they never reached the weight of controls animals. (**b**) Atrogin-1 and MuRF1 E3 ligase transcript levels were significantly higher in muscles from *Smn*^*2B*/−^ mice compared to control counterparts (p ≤ 0.01 and p ≤ 0.05 respectively). However, this increase was attenuated in TSA-treated *Smn*^*2B*/−^ mice (p ≤ 0.01 and p ≤ 0.05 respectively). (**c**) TSA treatment of *Smn*^*2B*/−^ mice resulted in a decrease in the level of ubiquitinated proteins towards control levels. (**d**) Gabarapl1, CathepsinL and Bnip3 mRNA levels in muscles of *Smn*^*2B*/−^ mice were restored to control levels upon TSA treatment. (**e,f**) LC3-II and P62 protein levels in *Smn*^*2B*/−^ mice dropped to control levels upon TSA treatment. (N = 3 for all experiments except c (*Smn*^*2B*/−^ TSA N = 4); p ≤ 0.05 for *, p ≤ 0.01 for **, and p ≤ 0.001 for ***).

Trichostatin A, a pan HDAC inhibitor, has shown beneficial effects on atrophy in the *Smn*Δ*7* model mice^17^. It is unclear whether this benefit was due to increased SMN protein, due to chromatin changes affecting other protective pathways, or both. Moreover, observations in the Bricceno *et al*.^17^ study were limited to proteasomal degradation and overlooked autophagy. Here, we have used the *Smn*^*2B*/−^ model, which does not contain the human *SMN2* transgene, to address these questions further. We treated the mice daily with either DMSO or TSA (10 mg/kg) from P3 to P21. Strikingly, TSA administration effectively rescued all dysregulated parameters we had observed. TSA treated *Smn*^*2B*/−^ mice had a significant increase in body weight compared to DMSO treated *Smn*^*2B*/−^ mice (p ≤ 0.01), although not quite comparable to DMSO treated control mice (p ≤ 0.001), reminiscent of other studies^18^,^19^ (Fig. 4a). Both Atrogin-1 and MuRF1 transcript levels at P21 were brought back to basal levels upon TSA treatment (Fig. 4b). This was also associated with a decrease in the levels of ubiquitinated proteins (Fig. 4c). The autophagosomal pathway was modulated in a similar fashion. The mRNA expression levels of Gabarapl1, CathepsinL and Bnip3 in muscles of TSA treated *Smn*^*2B*/−^ mice were comparable to those of control mice (Fig. 4d). Additionally, LC3 and P62 protein levels were back to normal (Fig. 4e,f). These results are consistent with our previous published findings where TSA treatment rescued skeletal muscle fiber size^11^,^19^. Altogether, it suggests that the beneficial effect of TSA on *Smn*^*2B*/−^ mice is likely occurring through a correction of the atrophy pathways in skeletal muscles, and is independent of SMN induction.

### FoxO transcription factors are induced in muscles of *Smn* ^*2B*/−^ mice

**Figure 5:**
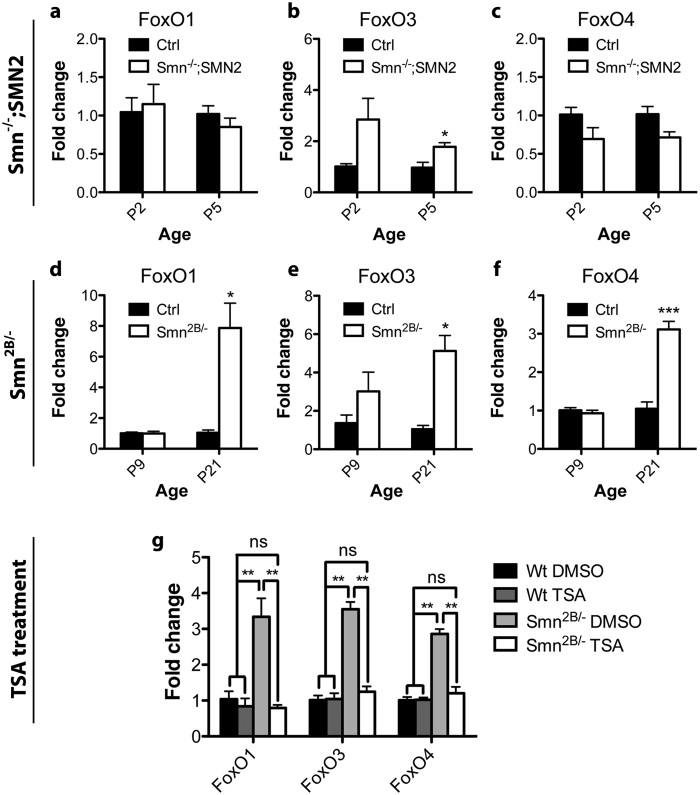
Alterations in the expression of the FoxO family of transcription factors are present in skeletal muscles of symptomatic stage *Smn*^*2B*/−^ mice and are reversed upon TSA treatment. (**a–c**) FoxO mRNA levels are generally not altered in muscles from *Smn*^*−*/*−*^*; SMN2* mice, except for FoxO3 which is slightly increased at symptomatic stage (P5). (**d–f**) In contrast, muscles from *Smn*^*2B*/−^ mice show a significant increase in the levels of FoxO1, FoxO3 and FoxO4 transcripts at the symptomatic stage (P21) in comparison to controls (p = 0.024, 0.012, and 0.00032, respectively). (**g**) FoxO mRNA levels in muscles of *Smn*^*2B*/−^ mice dropped to control levels upon TSA treatment. (N = 4 for a–f, N = 3 for g; p ≤ 0.05 for *, p ≤ 0.01 for **, and p ≤ 0.001 for ***).

Our observation of autophagosomal pathway involvement in muscles of *Smn*^*2B*/−^ mice associated with its significant improvement upon TSA administration led us to investigate new potential regulators of atrophy in SMA. Overexpression of MyoG and its targets were previously shown to be corrected by TSA administration^17^. However, MyoG does not regulate autophagosomal pathways and, thus, our results suggest at least one additional player in the control of atrophy in SMA. The FoxO transcription factor family of proteins are ideal candidates. They are part of the IGF1/PI3K/AKT pathway and they are capable of controlling both the proteasomal and autophagolysosomal genes in skeletal muscles^27^. Increased transcription of these factors has previously been reported on numerous occasions following different atrophic stimuli^14^,^35^,^36^,^37^. Additionally, FoxO1 is transcriptionally regulated by itself and by FoxO3, while FoxO4 is transcriptionally regulated by FoxO3^38^. Thus, we assessed mRNA levels of FoxO1, FoxO3 and FoxO4 in both the *Smn*^*−*/*−*^*; SMN2* and *Smn*^*2B*/−^ model mice. We suspected that *Smn*^*−*/*−*^*; SMN2* mice would not show any induction of these factors since autophagosomal transcriptional targets of FoxOs were not up-regulated in muscles of these mice (Fig. 1e–h). We did not observe any changes in FoxO1 and FoxO4 transcript levels (Fig. 5a,c). A small, but significant (p = 0.016), almost 2 fold increase in FoxO3 transcript levels in the symptomatic *Smn*^*−*/*−*^*; SMN2* hindlimb muscles was observed (Fig. 5b). In contrast, mRNA levels for FoxO1, FoxO3 and FoxO4 were significantly elevated by at least 3 fold in *Smn*^*2B*/−^ hindlimbs at P21 (p = 0.024, 0.012, and 0.00032, respectively) (Fig. 5d–f). Since we were able to rescue the autophagosomal abnormalities seen in the *Smn*^*2B*/−^ model upon TSA administration, we assessed whether or not expression of FoxO transcripts would be similarly restored to normal levels. Indeed, transcript levels of all three FoxO transcription factors reverted back to control levels in TSA treated *Smn*^*2B*/−^ hindlimb muscles (Fig. 5g). Altogether, this data provides evidence that FoxO factors are induced to trigger autophagosomal degradation in muscles of *Smn*^*2B*/−^ mice, and further that this induction can be reversed by TSA.

### The FoxO pathway is induced in cardiac muscle of *Smn* ^*−*/*−*^ *; SMN2* mice

Unlike skeletal muscle, cardiac muscle is innervated by autonomic nerves. Thus, cardiac muscle should not experience denervation and consequently, should not undergo atrophy in the context of SMA. Nonetheless, the hearts of *Smn*Δ*7* mice display smaller fiber size, which likely contributes to cardiac remodeling^24^. However, it remains unknown as to why this happens. Similar to skeletal muscles, FoxO transcription factors are also expressed in cardiac muscles^39^. FoxO3 induces Atrogin-1 and MuRF1 transcription, and counteracts cardiac hypertrophy in multiple pathological settings^39^. FoxO1 and FoxO3 also can induce autophagy in cardiac muscle^40^. Additionally, mouse models with constitutively active FoxO3 show decreased heart weight explained by individual cardiomyocyte size reduction^41^. Thus, we were intrigued whether atrophy processes in skeletal muscles analysed in this study might also be involved in cardiomyocyte fiber size reduction observed in SMA^24^. We therefore repeated the same experiments for both *Smn*^*−*/*−*^*; SMN2* and *Smn*^*2B*/−^ whole hearts at symptomatic age.

**Figure 6:**
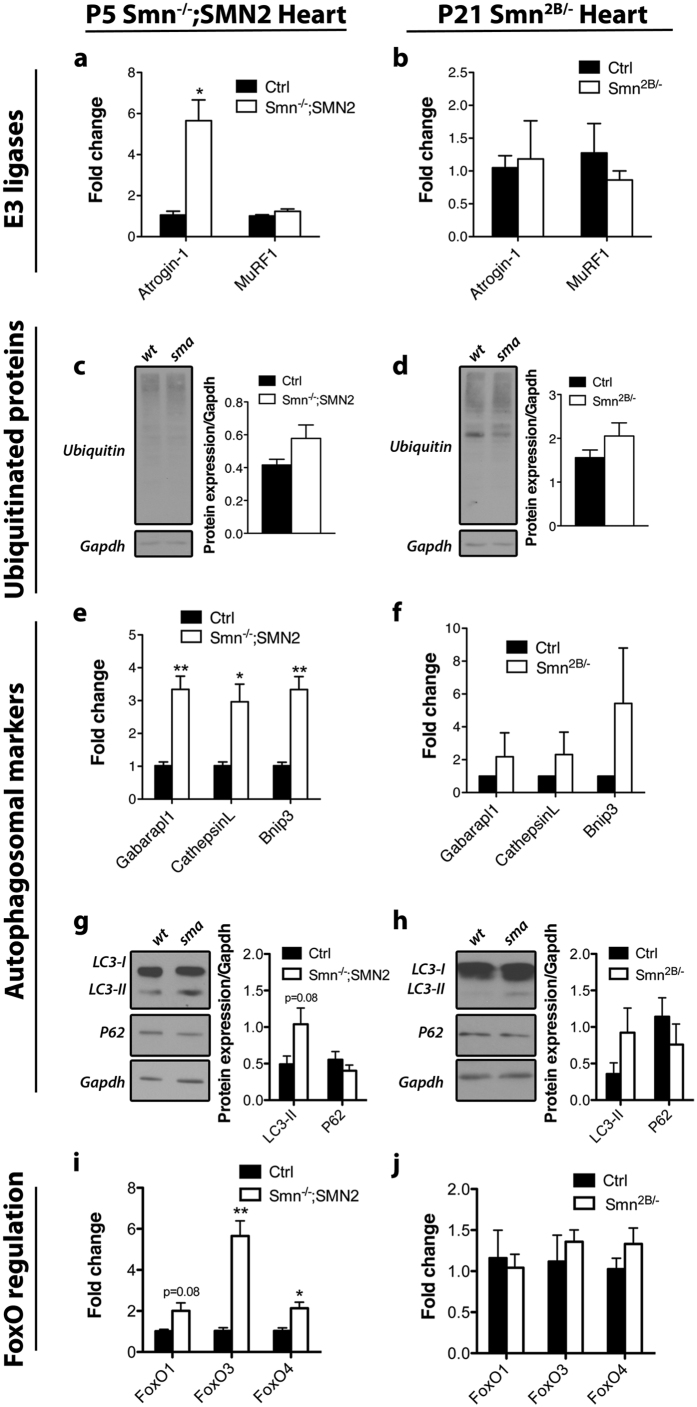
Alterations in the expression of the FoxO family of transcription factors and its targets in cardiac muscle of symptomatic stage SMA model mice are restricted to the severe *Smn*^*−*/*−*^*; SMN2* strain. (**a,c**) Increased Atrogin-1 (p = 0.018), but not MuRF1, mRNA levels are observed in *Smn*^*−*/*−*^*; SMN2* hearts at P5. However, this is not accompanied by an increase in the level of ubiquitinated proteins. (**b,d**) The levels of Atrogin-1 and MuRF1 mRNA are not altered in hearts from P21 *Smn*^*2B*/−^ mice. Similarly, there was no change in the level of ubiquitinated proteins. (**e,f**) Gabarapl1, CathepsinL and Bnip3 mRNA levels are elevated in P5 *Smn*^*−*/*−*^*; SMN2* hearts (p = 0.0076, 0.033, and 0.0075, respectively) but not in P21 *Smn*^*2B*/−^ hearts, although there was a trend towards an increase in all autophagosomal markers. (**g,h**) No change in LC3-II or P62 protein levels in the hearts of either model. (**i,j**) An increase in the mRNA level of some of the FoxO transcriptional factor family is observed in hearts from P5 *Smn*^*−*/*−*^*; SMN2* mice (FoxO3, p = 0.0069 and FoxO4, p = 0.025) but not in hearts from P21 *Smn*^*2B*/−^ mice. (N = 4 for all experiments; p ≤ 0.05 for * and p ≤ 0.01 for **).

Our analysis of the hearts of *Smn*^*−*/*−*^*; SMN2* mice showed that Atrogin-1 mRNA levels were increased (p = 0.018) whereas those of MuRF1 were comparable to control mice (Fig. 6a). This was not associated with elevation in the levels of ubiquitinated proteins (Fig. 6c). This suggests that Atrogin-1 may have some other role in cardiac muscle. Furthermore, although we did not observe any changes in expression of the autophagosomal markers in the hindlimb muscles of *Smn*^*−*/*−*^*; SMN2* mice, we did observe significant increased transcript levels of Gabarapl1, CathepsinL and Bnip3 in the heart (p = 0.0076, 0.033, and 0.0075, respectively) (Fig. 6e). Further, we observed a trend toward increased protein levels for Bnip3 and Gabarapl1 in hearts from P5 *Smn*^*−*/*−*^*; SMN2* mice (see Supplementary Fig. S1). Lipidation of LC3-I to LC3-II was increased but did not reach statistical significance, and P62 remained relatively unchanged (Fig. 6g). Finally, we noted that FoxO3 and FoxO4 transcript levels were significantly elevated (p = 0.0069 and 0.025, respectively) while FoxO1 transcript levels were elevated but did not reach statistical significance (p = 0.079) (Fig. 6i). Together, these results suggest that some type of autophagy, whether contributing to atrophy or other unknown processes, might be occurring in the hearts of the severe mice and could be controlled by FoxO transcription factors.

In marked contrast, the *Smn*^*2B*/−^ mice exhibit a different molecular profile in cardiac muscles. Proteasomal E3 ligase Atrogin-1 and MuRF1 mRNA levels remained unchanged, which was associated with stable protein ubiquitination (Fig. 6b,d). Interestingly, while the autophagosomal markers assessed in skeletal muscles provided solid evidence for autophagy, we only observed a trend towards induction for Bnip3, but not Gabarapl1 and CathepsinL, in P21 *Smn*^*2B*/−^ hearts (Fig. 6f). LC3-II and P62 were similar to control (Fig. 6h). In addition, FoxO transcript levels remain unchanged (Fig. 6j). Therefore, the *Smn*^*2B*/−^ heart appears relatively spared in contrast to its *Smn*^*−*/*−*^*; SMN2* counterpart. We further analysed the impact of TSA administration of *Smn*^*2B*/−^ mice on hearts at P21, and its potential effect on Atrogin-1 and the autophagosomal markers. We observed no difference in mRNA expression in Atrogin-1, Gabarapl1 and CathepsinL in DMSO and TSA-treated control and *Smn*^*2B*/−^ mice (see Supplementary Fig. S2). However, there was a significant increase in Bnip3 mRNA transcript levels in the hearts of DMSO-treated *Smn*^*2B*/−^ mice compared to wild type (see Supplementary Fig. S2), which confirms the trend observed in our initial experiment. Reminiscent to the muscle, TSA treatment of symptomatic *Smn*^*2B*/−^ mice restored Bnip3 transcripts back to control levels in the hearts of these mice (see Supplementary Fig. S2).

## Discussion

Atrophy is one of the pathological hallmarks of SMA. To date, a few studies have tried to elucidate the molecular mechanisms underlying the process of atrophy in SMA^17^,^20^. The focus of these studies has been mainly on MuRF1 and Atrogin-1 and their modulation by MyoG^17^. MyoG-dependent induction of these two E3 ligases is unlikely to be solely responsible for all the atrophy processes occurring in SMA muscles. For example, myogenin or HDAC knockout mice provide only partial resistance to muscle atrophy and overexpression of MyoG is not sufficient to trigger atrophy^15^. Additionally, the role of autophagolysosomal degradation in neurogenic atrophy is well established, but has not been studied in SMA^21^,^23^,^42^.

Here, we have focused on the characterization of the FoxO pathway in muscles from SMA model mice. The FoxO transcription factors are critical players in muscle atrophy, being required for the induction of both proteasomal degradation and autophagosomal degradation in various catabolic states, including neurogenic atrophy^21^,^22^,^23^,^42^. Furthermore, in FoxO knockout mice, muscle tissue is partially spared following denervation-induced atrophy and completely spared following fasting-induced atrophy^21^. Interestingly, both FoxO factors and many of their proteasomal-related and autophagosomal-related gene targets were induced post-symptomatically in skeletal muscles of *Smn*^*2B*/−^ mice. In addition, the detection of autophagic and lysosomal vacuoles strongly supports the involvement of autophagy mediated by FoxO transcription factors in skeletal muscles of *Smn*^*2B*/−^ mice. This does not preclude the role that MyoG may be playing in expression of the proteasomal E3 ligases MuRF1 and Atrogin-1 in *Smn*^*2B*/−^ mice. With respect to this, we have previously shown MyoG to be elevated in *Smn*^*2B*/−^ mice at symptomatic age^11^.

Identification of other catabolic mechanisms such as autophagy might offer an explanation for the inefficiency of MuRF1 and Atrogin-1 knockout *Smn*Δ*7* mouse models to gain weight or correct the muscle fiber size defect^20^. Whether autophagy is present in the *Smn*Δ*7* model mice remains to be demonstrated. Nevertheless, the importance of other catabolic mechanisms in maintaining atrophy is very likely. In fact, MUSA and SMART are two new E3 ligases recently discovered that are under the control of FoxO family of transcription factors^21^. These new E3 ligases contribute to fasting and neurogenic atrophy^21^. Targeting a transcription factor, such as FoxO, that controls expression of multiple proteins involved in protein breakdown may yield a better outcome in preventing atrophy than targeting downstream effectors, such as Atrogin-1 and MuRF1. Furthermore, the traditional individual Atrogin-1 and MuRF1 knockout mice have incomplete sparing upon denervation and was subtle in the first 7 days after denervation^25^. The short lifespan of the SMA models, including the S*mn*Δ*7* model (lifespan of ~14 days; loss of motor neurons at ~P9^43^), might not allow us to see the expected muscle sparing upon Atrogin-1 knockout. Indeed, this is what was observed by Iyer *et al*.^20^. It should also be stressed that weight and fiber size in the context of muscle studies in SMA might not be adequate since delayed myogenesis and atrophy simultaneously occurs in SMA muscles^11^,^12^,^44^. Impaired myogenesis is likely a key player in reduced fiber size and mouse weight. Consequently, it could mask or negate improvements that would be seen when modulating atrophy in SMA muscles.

The modulation of the IGF1 pathway as a means for possible therapy has also yielded modest results in the SMA field^45^,^46^,^47^. The first attempts aimed at inhibiting modulators of the IGF1 pathway, myostatin and its antagonist follistatin, with the hope that it would correct muscle fiber mass in SMA. The use of multiple strategies to inhibit myostatin resulted in transient increase in muscle mass, modest increase in lifespan, and unclear results in motor function tests^45^,^46^. In addition, targeting directly IGF1 by muscle-specific overexpression mimicked closely the results of earlier studies on myostatin and follistatin, albeit with slightly better outcomes on increased fiber size^47^.

Since SMA is a spectrum disorder, we took advantage of two mouse models to understand whether pathophysiologic mechanisms would be different. In fact, two variables may affect pathophysiology - the absolute level of SMN protein (severity-dependent) and the lifespan of the organism (time-dependent). For example, we have previously obtained different molecular patterns of impaired myogenesis in the *Smn*^*−*/*−*^*; SMN2* and *Smn*^*2B*/−^ mice that appeared to be time-dependent^11^. Microarray data from a cohort of type 1 and type 3 patients also demonstrated differences in the molecular profile of atrophy/hypertrophy^33^. Here, we provide evidence that the severity of disease in the two mouse models presents with different molecular patterns of atrophy induction. Strikingly, we observed that only the symptomatic *Smn*^*2B*/−^ skeletal muscles induce expression of the autophagosomal markers while the symptomatic *Smn*^*−*/*−*^*; SMN2* muscles do not. Consistent with this observation, FoxO transcription factor expression was induced in *Smn*^*2B*/−^ skeletal muscles but not in *Smn*^*−*/*−*^*; SMN2* muscles. Given the lifespan difference between the *Smn*^*−*/*−*^*; SMN2* and the *Smn*^*2B*/−^ mice, it is possible that *Smn*^*−*/*−*^*; SMN2* mice don’t live long enough for FoxO pathways to be induced. Indeed, the slight rise in FoxO3 transcripts in the *Smn*^*−*/*−*^*; SMN2* muscles lead us to think that *Smn*^*−*/*−*^*; SMN2* mice would potentially follow a similar protein degradation process if they could live longer. Interestingly, we also observe very different profiles in the atrophic markers of the heart tissues in the *Smn*^*−*/*−*^*; SMN2* and *Smn*^*2B*/−^ mice. However, this time the difference is in the opposite direction and appears to be dependent on severity and not time. While we see alterations in the expression of Atrogin-1, autophagosomal genes and some FoxO genes in the hearts of *Smn*^*−*/*−*^*; SMN2* mice, the *Smn*^*2B*/−^ hearts only display significant elevation of Bnip3. Interestingly, this altered expression in autophagosomal genes in *Smn*^*−*/*−*^*; SMN2* hearts was not observed in *Smn*^*−*/*−*^*; SMN2* skeletal muscles. In the same way, *Smn*^*2B*/−^ skeletal muscles displayed autophagic pathway induction while the *Smn*^*2B*/−^ heart muscles mostly did not. Therefore, cardiac muscle and skeletal muscle show rather divergent but intriguing pathologies in the context of SMA. Altogether, these results stress two important points: extrapolation of pathophysiology claims should be done cautiously between SMA animal models of differing severity, and as patients live longer because of new pharmacologic treatment strategies, new pathophysiologic problems may arise.

Utilization of the HDAC inhibitor TSA has shown beneficial effects in SMA preclinical studies^11^,^18^,^19^,^48^,^49^. However, it is unclear whether TSA benefit is through transcriptional induction of the *SMN2* transgene or through the epigenetic regulation of other genes^18^,^19^,^50^. Bricceno *et al*.^17^ showed that TSA could effectively reduce expression of E3 ligases MuRF1 and Atrogin-1 through HDAC4 inhibition in the *Smn*Δ*7* model mice^17^. To elucidate TSA mechanisms on atrophy in our study, we used the *Smn*^*2B*/−^ mouse, which doesn’t harbour the *SMN2* transgene. In this mouse model, it was previously shown that SMN protein levels remained unchanged upon TSA treatment^19^. Here, TSA reduced FoxOs transcripts with a consequent reduction in the expression of all downstream FoxO effectors. Therefore, we conclude that TSA benefit in *Smn*^*2B*/−^ mice appears to be mediated by changes in the expression of genes other than SMN to alleviate atrophy in the mice. It is also possible that TSA has an effect on other cell types, such as neurons, which may negate atrophy at the earliest stages. However, the effect of TSA on motor neuron survival was negligible in two different mouse models of SMA^18^,^19^. In a recent study, TSA improved innervation status of denervated muscles, however, many endplates remained partially denervated or fully denervated^48^. We also can’t rule out the possibility that TSA could have a positive impact on FoxO repression. In fact, increased acetylation of FoxO3 results in cytosolic localization and inactivity, and this can be reversed by HDAC1^36^,^37^,^51^. Other studies have also shown that TSA treatment resulted in cytosolic FoxO1 and FoxO3 localization leading to reduce expression of FoxO-induced gene targets including Atrogin-1, MuRF1, and Lc3B^51^.

The heart has received particular attention as a number of case studies reported cardiac defects in SMA patients^52^,^53^,^54^. Preclinical studies have investigated SMA cardiac function and anatomy^24^,^49^,^55^,^56^. Three main recurrent findings from these studies are small but proportionate heart size, bradycardia and abnormal heart remodelling^24^,^49^,^55^,^56^. In our study, we identify induction of E3 ligase Atrogin-1 and multiple genes involved in autophagy in hearts from the severe *Smn*^*−*/*−*^*; SMN2* mice but not in hearts from the less severe *Smn*^*2B*/−^ mice. Interestingly, our data could bridge gaps in what has been previously identified in SMA hearts. Firstly, it is possible that the proteasomal and the autophagosomal systems both contribute to reduced fiber size of individual cardiomyocytes^24^ and consequently to the smaller SMA hearts. Secondly, reminiscent to our study, the presentation of the thinner interventricular septum in the severe and *Smn*Δ*7* model is severity-dependent^55^. In support of this, most case reports of cardiac defects in humans are in type I SMA patients and rarely in type II and III^52^,^53^,^54^. Lastly, mice with constitutively active cardiac FoxO3 expression exhibit smaller hearts, reduced cardiomyocyte size, and reduced stroke volume and cardiac output^41^. This resembles findings in SMA hearts^24^,^56^. Further research is warranted to delineate whether the increased expression of FoxO transcriptional factors and autophagic genes is pathological or protective in the context of SMA.

In summary, we demonstrate that different mouse models of SMA display different molecular signatures of atrophy induction in both skeletal and heart muscles. Further, we show that TSA likely alleviates atrophy in an SMN-independent manner by targeting changes in expression of genes involved in the FoxO pathway. Finally, we show that hearts from severe SMA mice are potentially in a catabolic state, which may contribute to their abnormal functioning. Gaining a better comprehension of non-neuronal contributions, differences in commonly used mouse models, and the mode of action of HDAC inhibitors will help in advancing our understanding of SMA pathogenesis and the development of novel therapeutic strategies.

## Materials and Methods

### Mouse Models

The *Smn*^*−*/*−*^*; SMN2* (Jackson Laboratory) and *Smn*^*2B*/−^ mouse lines were housed at the University of Ottawa Animal Facility and cared for according to the Canadian Council on Animal Care. *Smn*^+/−^ mice were crossed to *Smn*^*2B/2B*^ mice to obtain *Smn*^*2B*/+^ and *Smn*^*2B*/−^ animals^9^. Tissues were harvested from pre-symptomatic and symptomatic *Smn*^*−*/*−*^*; SMN2* mice at P2 and P5, respectively. Pre-symptomatic tissues were collected at P9 while symptomatic tissues were dissected at P21 for the *Smn*^*2B*/−^ mouse model.

### TSA administration

TSA (10 mg/kg) was administered daily as previously described^19^. The treatment period was modified to P3-P21 to ensure maximal benefit^57^. Mice were sacrificed and dissected after the last TSA/DMSO injection at P21.

### Immunoblotting

Total protein lysate from SMA model mice and control animals was collected by either crushing liquid nitrogen frozen tissue with a pre-cooled mortar/pestle, and mixing muscle powder with RIPA lysis buffer (Cell Signaling) or homogenization in RIPA lysis buffer (Cell Signaling). Protein concentrations were determined using the Bradford method (Bio-Rad). Protein extracts were subjected to sodium dodecyl sulfate polyacrylamide gel electrophoresis and examined by immunoblot, as previously described^58^. Primary antibodies used were as follows: glyceraldehyde-3-phosphate dehydrogenase (Gapdh, Abcam: ab9485 - 1:2500 and ab8245 - 1:10000 or 1:12000), LC3B (Abcam: ab48394 - 1:1000), Ubiquitin (BioLegend (646301): Clone P4D1 - 1:1500), P62/Sqstm1 (Abcam: ab56416 - 1:1000), Bnip3 (Abcam: ab10433 - 1:1000), Gabarapl1 (ab86487 - 1:500). Secondary antibodies used were horseradish peroxidase (HRP)-conjugated anti-mouse immunoglobulin G (IgG, Bio-Rad) and HRP-conjugated anti-rabbit IgG (Bio-Rad) or IRDye 680 or 800 (Li-Cor). Signals were detected using enhanced chemiluminescence (Thermo) for standard western blotting, while fluorescence western blotting was performed with Odyssey CLx (Li-Cor). Densitometric analysis was performed using either ImageJ software or Image Studio 4.0 software. Results were normalized to Gapdh levels. LC3 protein levels were analysed as suggested by the guidelines to monitor autophagy^30^.

### RNA isolation and reverse transcription-quantitative polymerase chain reaction (RT-QPCR)

Total RNA was extracted from mouse models of SMA and wild type controls using RNeasy kit (Qiagen) according to the manufacturer’s protocol. RNA concentrations were determined using a nanophotometer spectrophotometer (MBI Lab Equipment). RNA was reversed transcribed using the quantitect reverse-transcription kit (Qiagen) according to the manufacturer’s protocol. QPCR was performed in triplicate for each sample using primers targeting Atrogin-1, MuRF1, FoxO1, FoxO3, FoxO4, Gabarapl1, CathepsinL, Bnip3 and Gapdh. A complete list of primers is available in the supplementary material (See Supplementary Table S1). Each QPCR reaction contained 50 ng of cDNA, 2x SyBR Green JumpStart Taq ReadyMix for QPCR (Sigma Aldrich) or Evagreen SyBR (Biorad), RNase/DNase-free water and appropriate primers (100–200 nM) in a final volume of 25 μl. Two negative controls were included in every QPCR plate and consisted of water in lieu of cDNA. QPCR results were quantified using 2^−∆∆Ct^ method. Results were normalized to Gapdh as an internal control.

### Transmission Electron Microscopy

P21 *Smn*^*2B*/+^ and *Smn*^*2B*/−^ mice were anaesthetized and perfused transcardially with 5 ml of phosphate-buffered saline (PBS) followed by 10 ml of Karnovsky’s fixative (4% paraformaldehyde, 2% glutaraldehyde and 0.1 M sodium cacodylate in PBS, pH 7.4). TA muscles were collected and fixed overnight at 4 °C in Karnovsky’s fixative. After fixation, each TA muscle was cut under a stereomicroscope into straight segments of 1 mm length. All segments were subsequently washed twice in 0.1 M sodium cacodylate buffer for 1 hour and once for overnight at room temperature. TA segments were post-fixed with 1% osmium tetroxide in 0.1 M sodium cacodylate buffer for 1 hour at room temperature. Segments were then washed in distilled water three times for 5 min. Specimens were dehydrated twice for 20 min for each step in a graded series of ethanol from water through 30%–50%–70%–85%–95% ethanol and twice for 30 minutes in 100% ethanol, followed by twice for 15 min in 50% ethanol/50% acetone and twice for 15 min in 100% acetone. TA segments were infiltrated in 30% Spurr resin/acetone for 20 min and once for 15 hours (overnight), then in 50% Spurr resin/acetone for 6 hours and in fresh 100% Spurr resin for overnight. Spurr resin was changed twice a day for three days at room temperature. All infiltration steps were performed on a nutator. Segments were embedded in fresh liquid Spurr resin and oriented inside the mould and then polymerized overnight at 70 °C. Ultrathin sections (80 nm) were collected onto 200-mesh copper grids and stained with 2% aqueous uranyl acetate and with Reynold’s lead citrate. A little more than 130 electron micrographs per genotype were examined at different magnifications using a transmission electron microscope (Hitachi 7100).

### Statistical analyses

All graphs represent means ± standard error of the mean. A two-tailed two sample Student’s *t* test of unequal variance was performed using Microsoft Excel to compare the means of control and SMA groups. One-way ANOVA analysis was used to distinguish difference between treated and non-treated groups. The post-test used for the ANOVA was Bonferroni. Significance was set at p ≤ 0.05 for *, p ≤ 0.01 for **, and p ≤ 0.001 for ***.

## Additional Information

**How to cite this article**: Deguise, M.-O. *et al*. Differential induction of muscle atrophy pathways in two mouse models of spinal muscular atrophy. *Sci. Rep.* **6**, 28846; doi: 10.1038/srep28846 (2016).

## Supplementary Material

Supplementary Information
