# Supplementary material for: Differential induction of muscle atrophy pathways in two mouse models of spinal muscular atrophy
Source: Sci Rep. 2016 Jun 28;6:28846. doi: 10.1038/srep28846 (PMC4924104; doi:10.1038/srep28846)
Supplement: Supplementary Information [file srep28846-s1.pdf]

Differential induction of muscle atrophy pathways in two mouse models of spinal muscular atrophy

Marc-Olivier Deguise[1,2,3], Justin G. Boyer[1,2,4], Emily McFall[1], Armin Yazdani[1,2], Yves De Repentigny[1] and Rashmi Kothary[1,2,3,5,*]

[1]Regenerative Medicine Program, Ottawa Hospital Research Institute, Ottawa, Ontario, Canada K1H 8L6

[2]Department of Cellular and Molecular Medicine, University of Ottawa, Ottawa, Ontario, Canada K1H 8M5

[3]Centre for Neuromuscular Disease, University of Ottawa, Ottawa, Ontario, Canada K1H 8M5

[4]Present address: Cincinnati Children's Hospital Medical Center, Cincinnati, OH 45229, USA

[5]Department of Medicine, University of Ottawa, Ottawa, Ontario, Canada K1H 8M5

*Correspondence to: Rashmi Kothary; Ottawa Hospital Research Institute; 501 Smyth Road, Ottawa, Ontario, Canada K1H 8L6; Tel: (613) 737-8707; Fax: (613) 737-8803; Email: rkothary@ohri.ca

Supplementary Table S1: List of primers used in this study and their respective annealing temperatures.

Gene target	Forward primer	Reverse primer	Ta
**Atrogin-1**	CGTCTCACTTTCCCCTCAAG	GACTCCCAGCCATCCAATTAG	57
**MuRF1**	AGTGTCCATGTCTGGAGGTCGTTT	ACTGGAGCACTCCTGCTTGTAGAT	60
**GabarapL1**	CATCGTGGAGAAGGCTCCTA	ATACAGCTGGCCCATGGTAG	62
**Bnip3**	TTCCACTAGCACCTTCTGATGA	GAACACCGCATTTACAGAACAA	60
**CathepsinL**	GTGGACTGTTCTCACGCTCAAG	TCCGTCCTTCGCTTCATAGG	60
**FoxO1**	CAAAGTACACATACGGCCAATCC	CGTAACTTGATTTGCTGTCCTGAA	60
**FoxO3**	CCTCATCTCAAAGCTGGGTAC	GGTTTTCTCTGTAGGTCTTCCG	63
**FoxO4**	AAATGCCAGCCTCGGCCAGC	GGCATTCCTCCCAGTAACAG	60
**Gapdh**	TCGGTGTGAACGGATTTG	GGTCTCGCTCCTGGAAGA	62

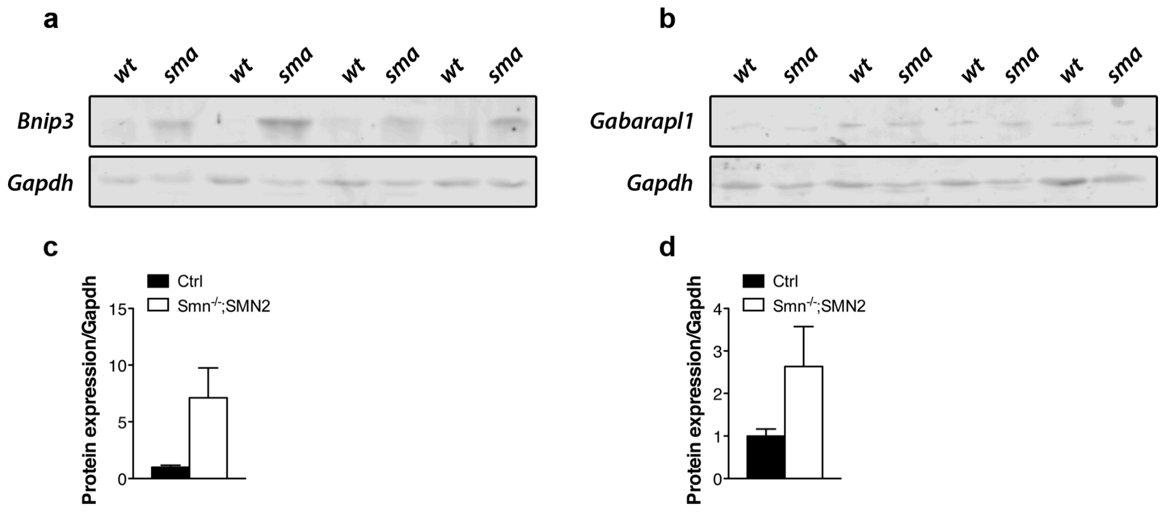

Supplementary Figure S1. ***Smn$^{-/-}$;SMN2* hearts show a trend toward increased Bnip3 and Gabarapl1 protein levels at P5.** (a) Western blot of Bnip3. (b) Western blot of Gabarapl1. (c) Quantification of Bnip3 protein expression showing a trend toward elevated protein levels compared to control. (d) Quantification of Gabarapl1 protein expression showing a trend toward elevated protein levels compared to control.

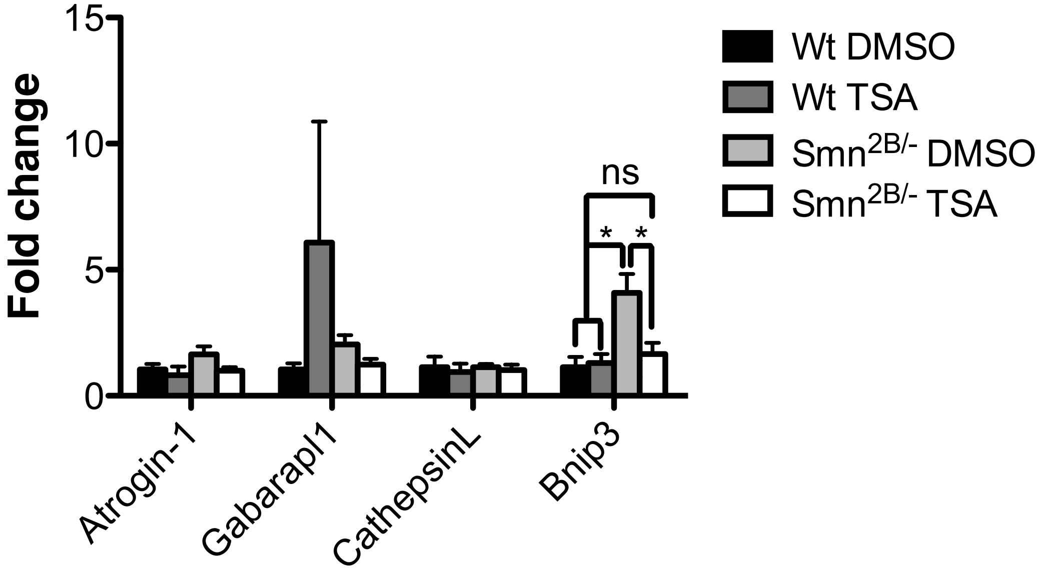

Supplementary Figure S2. **TSA administration to *Smn*$^{2B/-}$ mice restores Bnip3 expression in hearts to normal levels.** Atrogin-1, Gararapl1 and CathepsinL were unchanged as observed above. TSA treatment did not alter their expression. The increased expression of Bnip3 was effectively brought back to wild type control levels. (N=3 for all experiments; $p \leq 0.05$ for *)
